# Physicochemical and Microbiological Stability of the Extemporaneous Sildenafil Citrate Oral Suspension

**DOI:** 10.3797/scipharm.1505-08

**Published:** 2015-07-07

**Authors:** Attawadee Sae Yoon, Somchai Sawatdee, Chuthamas Woradechakul, Kridsada Sae Chee, Apichart Atipairin

**Affiliations:** 1 School of Pharmacy, Walailak University, Thasala, Nakhon Si Thammarat 80161, Thailand; 2 Drug and Cosmetic Research and Development Unit, Walailak University, Thasala, Nakhon Si Thammarat 80161, Thailand

**Keywords:** ASEAN guideline, Extemporaneous preparation, Sildenafil, Stability study, Suspension

## Abstract

Sildenafil is a potent and selective phosphodiesterase-5 inhibitor that is effectively used in the treatment of pulmonary arterial hypertension. In several countries, hospital pharmacists prepare the drug in an extemporaneous liquid preparation as there are no liquid formulations available for pediatric and adult uses. The purpose of this study was to evaluate the stability of an extemporaneous sildenafil citrate oral suspension for 90 days, according to the ASEAN guideline on stability studies of drug products. The results showed that the preparation was a white suspension with a sweet taste. It was a viscous and weakly acidic mixture with pseudoplastic behavior. The drug content was in the range between 99.23% and 102.23%, and the microbial examination met the general requirements throughout the study period. Therefore, the extemporaneously compounded sildenafil suspensions were physically, chemically, and microbiologically stable for at least 90 days when stored at 30° and 40°C. Furthermore, the in-use stability study showed that the preparations had acceptable attributes at least 14 days after the first-time use. This might provide an alternative option when the commercial suspension is unavailable.

## Introduction

**Fig. 1:**
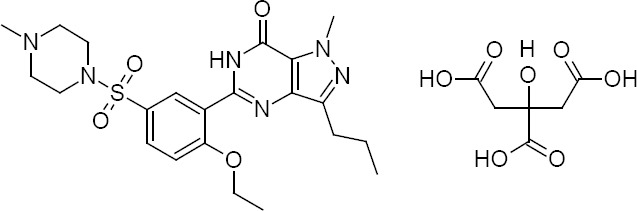
Chemical structure of sildenafil citrate.

Sildenafil citrate is chemically known as 5-[2-ethoxy-5-(4-methylpiperazine-1-sulfonyl)phenyl]-1-methyl-3-propyl-1,6-dihydro-7H-pyrazolo[4,3-d]pyrimidin-7-one citrate (Fig. 1). It is a potent and selective inhibitor of phosphodiesterase type 5 [1]. It inhibits the catalytic decomposition of cyclic guanosine monophosphate (cGMP) that results in the relaxation of smooth muscle cells of blood vessels. The drug has been used for the treatment of diverse medical conditions in cardiovascular diseases such as erectile dysfunction and pulmonary arterial hypertension (PAH) [2, 3].

Recently, several studies have demonstrated that sildenafil improves exercise capacity and hemodynamic parameters by significantly reducing pulmonary vascular resistance and mean pulmonary artery pressure [4, 5]. These reflect the efficacy and safety of the drug in the management of PAH by which the dose of 20 mg three times a day was approved for the treatment of adult patients who suffered from this disease [6]. Furthermore, a number of clinical trial studies were established to evaluate a potential use of sildenafil in infants and children [7, 8]. It was shown that the dose of 1 to 5 mg per kg three times daily showed an effective response in pediatric patients. Although it was an off-label or unlicensed drug, sildenafil met the clinical needs and was widely used in pediatric wards [9]. Nevertheless, it is only available in a tablet dosage form in several countries. Extemporaneous preparation of the drug in an oral liquid form is useful and suitable as it is a patient-specific weight-based dosage in a friendly preparation for children and adults who are unable to swallow tablets [10]. According to the United States Pharmacopeia, these aqueous preparations expire after 14 days if there is no stability test data [11]. Therefore, stability studies of such preparations are necessary to deliver the drug product to patients with quality, safety, and efficacy.

A previous study demonstrated the preparations of two extemporaneously compounded sildenafil citrate suspensions [12]. The preparations were made from a commercial tablet and found to be chemically stable throughout the 91 days of the study period when stored at 4°C and 25°C. However, there were no evidence-based examinations on the physical and microbiological stability to ensure product quality and consistency. In addition, there were two investigations to assess the oral liquid formulations of sildenafil. These studies used the active substance rather than the commercial tablet to prepare the drug in the solution and suspension dosage forms [13, 14]. Although it was impractical to directly use the pure chemical in some countries, there were advantages to make homogeneous preparations with a precise dose and avoid the potentially undesirable adverse effects of excipients. The results showed that all preparations were acidic mixtures and complied with the pharmacopoeia specification on microbial examination. In addition, the shelf life of these suspensions was 90 days, whereas the solutions were less stable in a range between 7 to 90 days when stored at 4°C and 25°C. An increase in the shelf-life of the suspension when compared to the solution was a result from the drug reservoir that made the interplay between the solubility and stability of the drug. However, the usage of these preparations was restricted by which it was recommended to keep the products refrigerated and use them within 14 days.

In this study, an extemporaneous compounding of sildenafil was prepared for an oral liquid suspension (2.5 mg/mL) from a generic 100 mg tablet only available in the hospital. This pre-formulated suspension could individually be taken by pediatric patients based on body weight. The short-term physical, chemical, and microbiological stability was evaluated when the drug product was exposed to a combination of high temperature and high humidity over the 90-day study period. It simulated the use of the product in practice with regards to both usage and storage conditions in tropical conditions, especially those in the ASEAN countries (30°C/75% RH and 40°C/75% RH) that was in accordance with the ASEAN guideline on the stability study of the drug products [15]. The obtained results would be valuable for the ASEAN hospital pharmacists for the preparation of the extemporaneous sildenafil suspension with acceptable quality for pediatric patients.

## Results and Discussion

### Physical Observations

After preparation, the extemporaneous compounding of sildenafil citrate was a white and fairly cloudy suspension with a sweet taste. Sucrose from simple syrup sufficiently hindered the bitter characteristic of the drug and improved child adherence. Furthermore, there were no detectable changes in color and taste in any samples during three months of storage at two controlled temperatures (30°C/75% RH and 40°C/75% RH). The suspended matter was some of the inert and insoluble excipients presented in the tablets. It precipitated as a function of time by which the longer the time of storage it had, the shorter the sedimentation height (Table 1). The precipitate of the preparation was loosely settled. Therefore, it could be simply redispersed by gentle shaking for 30 s. The sedimentation height at 30°C for 90 days was 0.25±0.04, and it used 5±2 reverse turns to resuspend the precipitate completely. The storage temperatures were not affected by the sedimentation height and ease of redispersion. These indicated that the appearance of the preparation was physically stable.

**Tab. 1:**
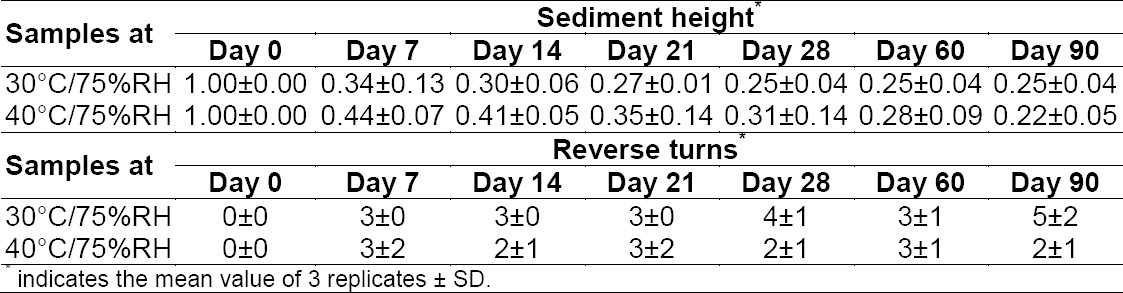
The sediment height and reverse turns of the extemporaneous preparations.

**Fig. 2:**
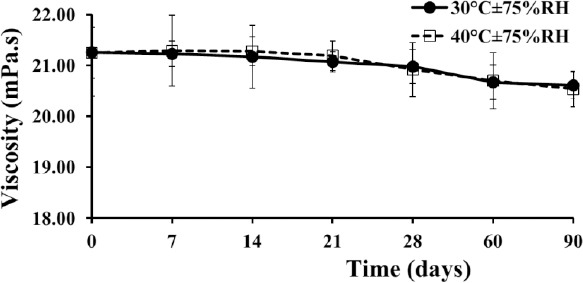
Viscosity of the extemporaneous sildenafil citrate suspensions. It is represented as the mean±SD of 3 replicates.

The viscosity of the freshly prepared extemporaneous suspension was 21.26±0.86 mPa.s. The presence of compounds such as methylcellulose, sucrose, and insoluble excipients in the preparation caused liquid resistance to flow. There were no statistically significant differences observed at the end of the storage period (Day 90) at both simulation temperatures (Fig. 2; *p-*value >0.05). This provided an important benefit in the dosification to ensure the consistency of the drug content in suspension, especially for the treatment of pediatric patients.

**Fig. 3:**
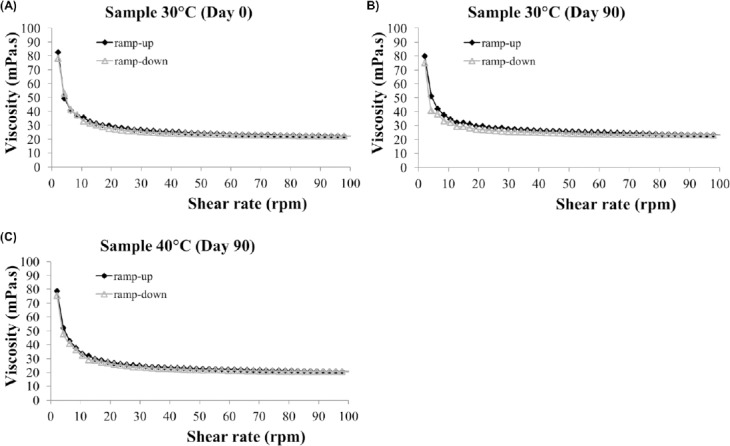
Rheogram of the extemporaneous sildenafil citrate suspensions. (A) was the drug product on Day 0 at 30°C, (B) and (C) were the drug products at 30°C and 40°C, respectively, on Day 90.

Furthermore, the study of rheology was used to evaluate the flow behavior of a liquid dosage form. It was found that the viscosity of the products decreased when the shear rate was increased from 0 to 100 rpm (Fig. 3). This phenomenon was defined as pseudoplasticity or shear thinning that was the most common rheological behavior of the heterogeneous pharmaceutical dosage forms [16]. In addition, when the shear stress was applied to the preparation for a finite time (1 min), the hysteresis loop of the viscosity was negligible. It indicated that this extemporaneous suspension did not show thixotropy characteristics. The results also demonstrated that the preparations remained unchanged in the rheological properties until the end of the study period (Fig. 3). It was consistent with previous studies, showing that methylcellulose exhibited pseudoplastic flow [17]. This could probably be from the shearing action on the linear polymer of methylcellulose presented in the preparation. As the shearing stress is increased, the unstructured molecules begin to align their long axes in the direction of the flow. This arrangement reduces the flow’s resistance that allows a decrease in the viscosity in response to the imposed shearing stress [17].

Taken together, there were no detectable changes in visual appearance, color, and taste of the extemporaneous suspensions over the 90 days of the study period. The preparations were easily resuspended and appeared to maintain constant viscosity and rheological behavior both at 30°C/75% RH and 40°C/75% RH. These indicated that the preparations were stable physically for 90 days.

### Chemical Stability

**Fig. 4:**
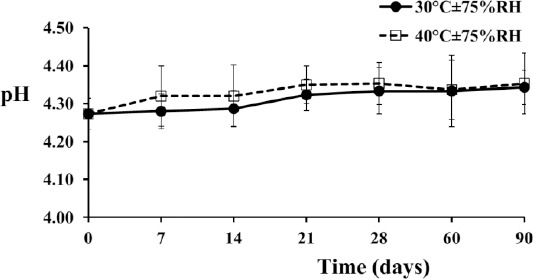
The pH profile of the extemporaneous sildenafil citrate suspension. It is represented as the mean±SD of 3 replicates.

The preparation had an initial pH value of 4.27±0.04, whereas that of the vehicle was 5.29±0.14. The weakly acidic characteristic of the product resulted from a tricarboxylate functional group of citrate salt. It provided a great advantage that allowed maximum solubility and permeability of sildenafil by means of being a cationic species in piperazine and forming an intramolecular hydrogen bond between an oxygen atom in sulfonamide and a proton in the basic nitrogen atom in piperazine, respectively [18–20]. The pH of the preparations was rather constant, and it was not statistically different over the time of storage (Fig. 4; *p-*value >0.05). Previous studies also showed that sildenafil was stable under acid- and basic-based hydrolysis with the percent recovery of more than 90% [21, 22]. In addition, the temperature of storage did not influence the pH as there were no statistically significant differences (*p-*value >0.05). The pH values of the product were in a range between 4.27±0.04 to 4.34±0.05 at 30°C and 4.27±0.04 to 4.35±0.08 at 40°C (Fig. 4).

**Fig. 5:**
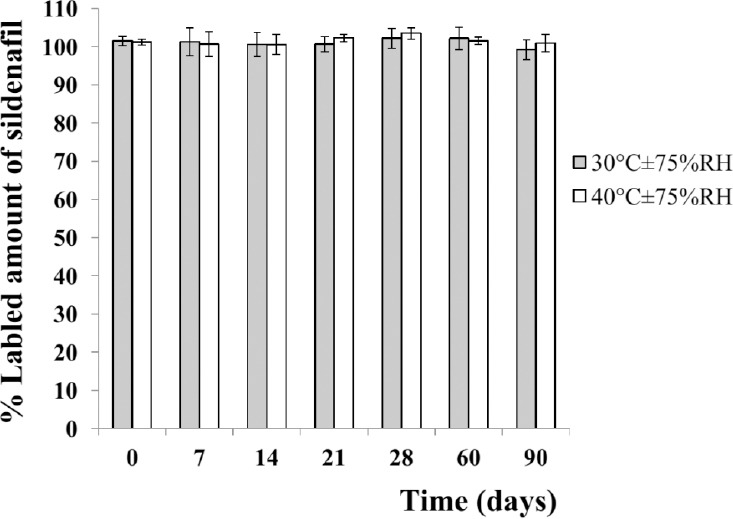
Percent labeled amount (%LA) of sildenafil of the extemporaneous preparations. It is represented as the mean±SD of 3 replicates.

The content of sildenafil citrate in the preparation was studied by an HPLC method. We performed method validation, according to the ASEAN guideline for the validation of an analytical procedure [21]. The method showed acceptable performance with high accuracy (100.18% recovery) and precision (< 2% RSD). Linearity was found in a concentration range of 0.01–60.00 µg/mL (r^2^ = 0.9999). The LOD and LOQ were 3.82 and 11.57 ng/mL, respectively. The proposed method was a stability-indicating procedure that could be used for the analysis of sildenafil in stability studies. It was shown that the percent label amount of sildenafil in the preparations was in a range of 99.23±2.66 to 102.23±2.60 at 30°C and 100.58±2.61 to 103.52±1.51 at 40°C (Fig. 5). There were no statistically significant differences observed after the end of the study period (90 days) at any temperature (*p-*value >0.05). Therefore, it indicated that the preparations were chemically stable as the drug contents were not less than 90% and not more than 110% of the labeled amount of sildenafil citrate [11]. These results were also consistent with our previous study that the extemporaneous sildenafil suspension was stable under various harmful conditions in the forced degradation studies such as acid and base hydrolysis, dry heat, and light [21]. However, the preparations were sensitive to oxidation as a function of the time and concentration of the oxidizing agent by which the oxidative reaction had taken place at the citrate anion, alkoxybenzenesulphonamide group, and piperazine ring [23].

### Microbial Examination

All preparations showed that the total count of bacterial, yeast, and mold was less than 10 colonies per mL (CFU/mL) in the storage conditions at 30°C and 40°C. In addition, there were no suspected colonies of *Escherichia coli* found in the selective media of any samples. The presence of 1% paraben concentrate as a preservative in the preparation exhibited effective antimicrobial activity. Thus, the extemporaneous suspensions were microbiologically stable for 90 days as they met the requirement of the acceptance criteria for non-sterile pharmaceutical products in the category of aqueous preparations for oral use.

### In-Use Stability

Because the extemporaneous suspensions were purposed for use as a multiple dose regimen for a short period of time, it was necessary to determine the physical, chemical, and microbiological stability of the preparations whether they retained the quality once the containers were opened under ambient conditions. It was found that the preparations were still white and cloudy suspensions with a sweet taste (Table 2). It was weakly acidic (pH 4.30±0.05 to 4.32±0.06). The viscosity value was 20.92±0.48 mPa.s, and the drug amount was 103.18%±3.88% after Day 14. In addition, the microbial growth was detected, but it was in the accepted specification. The in-use stability testing demonstrated that the preparations were stable and remained an appropriate quality, even when kept at room temperature (tropical climate zone) at least 14 days after the first-time use. However, extended storage time might be investigated to determine an exact beyond-use date.

**Tab. 2:**
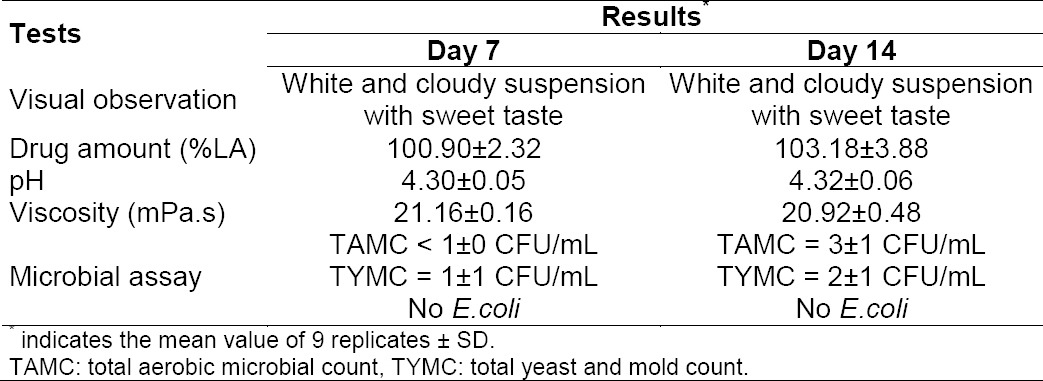
In-use stability studies of the extemporaneous preparations.

## Experimental

### Chemicals

Sildenafil 100 mg tablets (Sidegra^®^; lot no. A562751) were purchased from the Government Pharmaceutical Organization, Thailand. Methylcellulose-4000, methylparaben, propylparaben, and propylene glycol were supplied by the P.C. Drug Center Co. Ltd, Thailand. Sucrose was procured from Carlo Erba Reagents, Italy. Sildenafil citrate working standard (potency 99.40% as is) was received from Smilax Laboratories Limited, India. Soybean casein digest, Sabouraud dextrose, and MacConkey media were purchased from Becton Dickinson and Company, France. Other chemicals were analytical grade from Merck, Germany. All solvents used were HPLC grade from RCI Labscan, Thailand.

### Preparation of Extemporaneous Sildenafil Citrate Suspension

Twenty 100-mg sildenafil tablets were peeled out of the coated film, and they were thoroughly pulverized by a mortar and pestle. The vehicle was a mixture of 400 mL of 1% methylcellulose, 2% paraben concentrate, and 400 mL of simple syrup [21]. The powdered sildenafil tablet was mixed with a small portion of the vehicle to form a smooth paste. The remaining vehicle was added and mixed well to a final volume of 800 mL, using a graduated cylinder. The extemporaneous sildenafil suspension was prepared thrice. Sixty mL was then transferred to an amber glass bottle by which its labeling indicated the content of “the nominal strength of sildenafil 2.5 mg/mL” and “shake well before use”.

### Stability Study of the Extemporaneous Preparation

Stability of the extemporaneous sildenafil suspension was evaluated according to the ASEAN guideline on the stability study of the drug product [15]. Three different batches of the preparations were prepared, and 21 bottles of the drug product were stored at each temperature (30°C/75% RH and 40°C/75% RH). Physical, chemical, and microbiological examinations were performed in triplicate immediately after preparation (Day 0) and at Day 7, 14, 21, 28, 60, and 90 to define drug stability throughout its period of storage. The preparation was considered physically stable if those characteristics (appearance, pH, viscosity, and rheology) were not changed. The chemical stability of the extemporaneous preparation was defined by the drug content that contained not less than 90.0% and not more than 110% of the labeled amount of sildenafil [11]. All preparations were microbiologically stable if the total aerobic microbial count (TAMC) was less than 100 CFU/mL, the total yeast and mold count (TYMC) was less than 10 CFU/mL, and there was no suspected colony of *E. coli* in MacConkey agar.

### Appearance

The physical appearance properties were examined using visual observation of the samples stored at each condition. Those properties included color, taste, sedimentation height, and redispersibility. Sedimentation height was defined as a ratio of the height between the sediment and the mixture, whereas redispersibility represented the number of reverse turns to make a completely dispersed suspension.

### pH Measurement

The pH value of the samples was determined on each study day using a bench pH/mV meter Jenway-3510 (Bibby Scientific Limited, United Kingdom). Standard buffer solutions of pH 7.00, 4.00, and 10.00 were used to calibrate the device before each measurement. Three replicates of the samples were performed, and the results were shown as mean±standard deviation (SD).

### Viscosity and Rheological Behavior

A small portion of 6.7 mL of each sample was subjected to a Brookfield LVDV-III Ultra Rheometer equipped with a small sample adapter and TC-150SD water bath (Brookfield Engineering Laboratories, USA). The viscosity measurement was performed at 25°C, and the samples were subjected to a constant shear rate at 100 rpm for 1 min. It was determined in triplicate and expressed in units of milliPascal x seconds (mPa.s). In addition, the rheological behavior of the samples was obtained by recording the shear stress at given shear rates during the ramp-up period from 0 to 100 rpm, the constant shear rate period (100 rpm for 1 min), and the ramp-down period from 100 to 0 rpm. Control of the instrument and analysis of the data were carried out using Rheocalc Version 3.3 software. The results are presented as mean±SD.

### High-Performance Liquid Chromatography (HPLC) Analysis

The Ultimate 3000 instrument (Dionex Corporation, USA) was used for HPLC analysis of sildenafil content in the preparation. The method employed a C18 Inertsil^®^ ODS-3 column (4.6 × 250 mm; GL Sciences) that was kept at 25°C. The mobile phase was prepared from a mixture of 50% 0.2 M ammonium acetate buffer pH 7.0 and 50% acetonitrile. The flow rate was adjusted to 1.0 mL/min, and UV absorbance was detected at 245 nm. The injection volume was 10 µL, and each sample was analyzed in duplicate. The data was recorded and interpreted using Chromeleon 7 software. The proposed method was validated according to the ASEAN guideline for the validation of an analytical procedure [21].

### Preparation of Standard Solutions for HPLC Analysis

Sildenafil citrate working standard was accurately weighed and placed in a volumetric flask. It was dissolved, the volume was adjusted by the mobile phase, and it was used as a standard stock solution (250 µg/mL). It was diluted quantitatively with the mobile phase to obtain standard solutions in the range between 0.01 and 60.00 µg/mL.

### Preparation of Sample Solutions for HPLC Analysis

Extemporaneous sildenafil citrate suspension (about 2.5 mg/mL) was shaken well for 1 min. Five mL of the preparation was taken from the center of the container and placed into a 50-mL volumetric flask. It was mixed with the mobile phase, sonicated for 10 min, and finally the volume was adjusted to the mark with the mobile phase. Five mL of the sample aliquot was pipetted into a 50-mL volumetric flask and then diluted with the mobile phase. The sample solution was prepared in triplicate, and it was filtered through a 0.45 µm nylon-66 membrane filter before analysis.

### Microbiological Studies

Microbiological examinations were performed according to the United States Pharmacopeia monograph of non-sterile products [11]. Briefly, 10 mL of the samples were diluted with phosphate buffer at pH 7.2 to obtain a 1:10 dilution. Ten mL of the sample solution was pipetted into a sterile filtration apparatus. A membrane filter with a pore size of 0.45 µm was washed three times by 100 mL of the same diluent. To determine TAMC, the membrane filter was transferred to the surface of a soybean casein digest (SCD) agar. The plates were incubated at 35°C for 3 days. TYMC was performed by placing the membrane filter onto Sabouraud dextrose agar and incubating the plate at 25°C for 5 days. The colonies were counted and expressed as the average number of colony forming units (CFU) per mL of the sample. The presence of *E. coli* was examined by incubating a 10-fold dilution of the sample in SCD broth at 35°C for 24 h. One mL of the media was transferred into 100 mL of MacConkey broth and then incubated at 42°C for 24 h. It was streaked on MacConkey agar at 35°C for 48 h. If there is the growth of colonies, it indicates the possible presence of *E. coli*. The results of three replicates are shown as mean±SD of CFU.

### In-Use Stability

The extemporaneous preparations were assessed for in-use stability to determine the integrity of the multi-dose product once the container was opened. Three different batches of the preparations were studied for 14 consecutive days at room temperature by which the containers were shaken and opened thrice a day. One mL of the sample was removed from the preparation each time to simulate the real condition of use. The physical (visual appearance and viscosity), chemical (pH and drug content), and microbiological (TAMC, TYMC, and presence of *E.coli*) properties of the nine replicates of the preparations were monitored at Day 7 and 14.

### Statistical Analysis

The results are represented as the mean value of three independent experiments ± SD. They were analyzed by one-way analysis of variance (1-way ANOVA), followed by Student’s *t*-test. The level of significance was set at *p*<0.05. Turkey’s test was exploited for multiple comparisons to verify the statistical significance between means.

## Conclusion

Extemporaneously compounded sildenafil citrate 2.5 mg/mL oral suspension was physically, chemically, and microbiologically stable for 90 days when stored at 30°C and 40°C. The visual appearance, pH, and viscosity of all preparations did not significantly change. The rheological property of the drug product was pseudoplastic. It still retained a minimum of 99% of the labeled amount of the drug over the study period, and the microbial limits met the general requirements. In addition, the in-use stability study indicated that the preparations retained the product quality at least 14 days after the first-time use. These emphasize that the studied formulation was simple but stable, and it was convenient to prepare for pediatric patients.
